# Dietary *Nigella sativa* nanoparticles enhance broiler growth performance, antioxidant capacity, immunity, gene expression modulation, and cecal microbiota during high ambient temperatures

**DOI:** 10.1038/s41598-024-82725-9

**Published:** 2025-01-05

**Authors:** Ahmed M. Elbaz, Eman S. Ashmawy, M. A. A. Farahat, Ahmed Abdel‑Maksoud, Shimaa A. Amin, Zangabel S. Mohamed

**Affiliations:** 1 Animal and Poultry Nutrition Department, Desert Research Center, Mataria, Cairo Egypt; 2 Microbiology Department, Faculty of Agriculture, Ain Shams University, Cairo, Egypt; 3 Lecture of Poultry Production Animal Production Department, Faculty of Agriculture, Benha University, Benha, Egypt

**Keywords:** High ambient temperature, Broilers, Growth, Antioxidant, Immune response, Nigella sativa nanoparticles, Antimicrobial responses, Cytokines, Physiology, Zoology

## Abstract

Environmental heat stress causes significant economic loss in the poultry industry. Therefore, interest has increased in using feed additives to reduce the negative impacts of heat stress on the chickens and improve production performance. This study aimed to assess the effect of supplementing with *Nigella sativa nanoparticles* (Nano-NS) as an anti-stress and growth promoter in broiler diets under hot climatic conditions. A total of 375 male one-day-old Ross 308 chicks were randomly divided into a control group and four treatment groups (75 chicks/group). The first group fed a basal diet without additives, the second group fed a basal diet supplemented with avilamycin at 50 mg/kg, and the other groups fed a basal diet supplemented with 30, 40, and 50 mg/kg Nano-NS, respectively. Despite that feed intake was not affected, feed conversion ratio, body weight gain, and crude protein digestibility improved in broilers fed Nano-NS (*P* < 0.05) compared with avilamycin and the control groups. Adding Nano-NS led to an increase in the dressing percentage and the relative weight of the bursa of Fabricius and thymus. Serum high-density lipoprotein levels increased while total cholesterol and low-density lipoprotein concentrations decreased (*P* < 0.05) in broilers fed Nano-NS compared with control groups. Furthermore, Nano-NS supplementation significantly increased (*P* < 0.05) serum immunoglobulin (IgG and IgA), and superoxide dismutase (SOD) levels, while decreasing malondialdehyde (MDA), interleukin-6 (IL-6), and tumor necrosis factor-alpha (TNF-α) concentration. Moreover, there was a significant increase in the *Lactobacillus* population and a decrease (*P* < 0.05) in the *E. coli* and *C. perfringens* population in chicks fed Nano-NS. In the intestinal tissues, mucin 2 (MUC2) gene expression increased in chickens fed 50 mg/kg Nano-NS compared to other groups. It is concluded that adding Nano-NS (up to 50 mg/kg) reduced the negative effects of heat stress via enhancing growth performance, immune responses, and antioxidant status, modulating the microbial community structure, and increasing the expression of the MUC2 gene in broilers under high ambient temperature.

## Introduction

Environmental pressure is one of the greatest hurdles that negatively affect the poultry industry, especially global warming, which exposes the birds to heat stress (HS), leading to reduced well-being, performance, and health of the birds^1^. Additionally, recent genetic developments in broiler chicks have made them more sensitive to environmental changes because of the great metabolic rate resulting from selecting fast growth^2^. The increase in the ambient temperature than the thermoneutral zone of the bird leads to changes in the behavior of the bird in an attempt to cope with HS and lose excess heat through panting. Increased HS leads to an increase in panting and a decrease in the partial pressure of carbon dioxide and calcium availability leading to increasing pH value in the blood, which increases the risk of respiratory alkalosis^2,3^, as well as, the risk of oxidative stress, and that inhibits the function of both the digestive system and the immune^4^, which leads to gut dysfunction (inflammation), deterioration of bird’s health, decreased growth and utilization of nutrients, and an increase in mortality rate^5^. The small intestine is damaged by many factors, including pathogenic bacteria, unhealthy feeding conditions, and high ambient temperature^6,7^. HS causes an imbalance in the microbial content and inflammation in the intestines, which leads to a general weakness in the health and performance of the chicks^1,6^.

Recent studies have shown that feed additives effectively reduce the harmful effects of HS on birds, such as essential oils, probiotics, vitamins, trace minerals, etc^8–10^. , via regulating both immune-antioxidant status and microbiota-gut. *Nigella sativa* is widely used as an herbal medicine, that plays a useful role as a digestive stimulant in animals by stimulating the secretion of digestive enzymes (lipase and amylase) to stimulate feed digestion^11^. Additionally, it contains antimicrobial, anti-inflammatory, and antiviral attributes, in addition to its antioxidant properties (the active ingredients as nigellone and melatonin), it underpins bird immunology^12^. Several studies have shown the positive role of Nigella sativa supplements in enhancing growth performance by regulating adaptive immunity and controlling infectious diseases^13,14^. Furthermore, including *Nigella Sativa* seeds in the broiler’s diet positively affected blood profile, humoral immunity, and cell-mediated immunity^15^. Additionally, Nigella sativa had a positive effect on laying hens, as it increased egg production and quality^16^. *Nigella sativa* contains significant levels of protein (amino acids, 22.7%), carbohydrates (31.94%), fat (38.20%), and essential oils such as thymoquinoline and dithymoquinoline^17^.

Rapid development in the field of nanotechnology and the advantages of its associated products encouraged many scientific fields to use it. Nano compounds are characterized by their solid adsorption ability, efficacy in interacting with inorganic and organic materials inside the bird’s body due to their increased surface area and interaction with biological targets, and high catalytic efficiency^18,19^. In addition, nanocomposites have the ability to circulate into the blood and the internal organs and rapidly cross the small intestine^20^. Presumably, nanocomposites provide better bioavailability and interact better with other elements^21^. Besides, many studies have proven the success of using nanotechnology in poultry feed such as zinc oxide nanoparticles, selenium nanoparticles, etc^22,23^. More studies are needed to clarify the effects of nanoparticles of *Nigella Sativa* in alleviating the harmful impacts of HS in broiler chicks. From that, we hypothesized that adding *Nano-Nigella sativa* in broiler feed may play an important role as an effective alternative to antibiotics, in addition to reducing the impacts of HS on the chickens. Therefore, this study aimed to assess the effects of adding Nano-NS on growth, nutrient digestibility, blood metabolites, immune responses, antioxidant status, microbial community structure, and MUC2 gene expression in chickens exposed to environmental HS.

## Results

### Productive performance indices

Table 1 presented that feeding broilers with diets supplemented with Nano-NS has positive effects (*P* < 0.05) on growth performance. During the starter and grower period, there was a noticeable improvement in BWG and FCR in chickens fed 50 mg/kg Nano-NS compared to the other groups. Furthermore, during the overall period, the improved BWG in chickens fed 40 and 50 mg/kg Nano-NS (*P* < 0.05) compared to the rest of the groups, while FCR was enhanced in chickens fed 50 mg/kg Nano-NS compared to other groups. However, FI and mortality rates were not affected between the experimental groups during the different experimental periods. However, there was a significant improvement in EPEF with increasing Nano-NS levels in the experimental diet. Table 2 presented that feeding broilers with diets supplemented with Nano-NS had no effects (*P* < 0.05) on carcass characteristics, including the relative weight of thigh, breast, liver, and abdominal fat, except for dressing percentage that increased (*P* < 0.05) in broilers fed 50 mg/kg Nano-NS compared to other groups.

**Table 1:** Effect of supplementation of Nano-NS on growth performance of broilers under high ambient temperature.

Parameter	CON	AVI	Nano-NS_1_	Nano-NS_2_	Nano-NS_3_	SEM	*P*-value
**BWG, g.bird.d** ^− 1^							
1–21 d	35.34^c^	37.71^b^	37.32^b^	38.05^b^	40.90^a^	15.06	0.015
22–35 d	55.81^c^	57.68^b^	57.66^b^	59.51^a^	60.34^a^	19.69	0.020
1–35 d	45.59^c^	46.87^b^	46.66^b^	47.84^a^	48.68^a^	26.94	< 0.001
**FI**,** g.bird.d**^**− 1**^							
1–21 d	45.62	46.24	45.57	46.20	46.38	21.44	0.284
22–35 d	136.2	136.1	135.2	136.6	136.1	38.16	0.125
1–35 d	81.83	82.18	81.37	82.34	82.26	46.18	0.503
**FCR**,** g feed. g gain**^**− 1**^							
1–21 d	1.290^a^	1.225^b^	1.221^b^	1.213^bc^	1.191^c^	0.072	0.001
22–35 d	2.441^a^	2.360^b^	2.345^b^	2.296^c^	2.255^d^	0.075	< 0.001
1–35 d	1.794^a^	1.753^b^	1.744^b^	1.722^c^	1.689^d^	0.042	< 0.001
Mortality %	5	5	5	4	5	-	-
EPEF	248	260	261	273	281	-	-

Means for probiotic main effect within the same column differ significantly (*P* < 0.05), LBW; live body weight, BWG; body weight gain, FI; feed intake, FCR, feed conversion ratio, CON; basal diet without added, AVI; basal diet with avilamycin, Nano-NS1; basal diet with 30 mg/kg Nigella Sativa Nanoparticles, Nano-NS2; basal diet with 40 mg/kg Nigella Sativa Nanoparticles, Nano-NS3; basal diet with 50 mg/kg Nigella Sativa Nanoparticles, EPEF; European Production Efficiency Factor.

**Table 2:** Effect of supplementation of Nano-NS on the carcass traits (%) and nutrient digestibility (%) of broilers under high ambient temperature.

Item	parameter	CON	AVI	Nano-NS_1_	Nano-NS_2_	Nano-NS_3_	SEM	*P*-value
Carcass traits	Dressing	75.6^c^	78.7^b^	77.9^bc^	79.2^b^	80.4^a^	0.518	0.001
	Breast	23.8	24.1	24.3	24.4	24.6	5.162	0.126
	Thigh	16.1	15.9	16.2	16.3	16.2	2.351	0.094
	Liver	3.41	3.37	3.51	3.35	3.45	0.085	0.038
	A. fat	4.28	4.41	4.35	4.38	4.24	0.201	0.071
Nutrient digestibility	DM	73.6^c^	75.3^b^	75.4^b^	77.1^a^	77.8^a^	0.132	0.012
	CP	65.4^c^	66.8^bc^	66.4^bc^	68.5^b^	70.1^a^	0.091	0.001
	CF	57.6	56.9	57.2	58.0	57.8	0.135	0.102

^a–b^ Means with different superscripts within the same row differ significantly (*P* < 0.05); SEM standard error of means, CON; basal diet without added, AVI; basal diet with avilamycin, Nano-NS1; basal diet with 30 mg/kg Nigella Sativa Nanoparticles, Nano-NS2; basal diet with 40 mg/kg Nigella Sativa Nanoparticles, Nano-NS3; basal diet with 50 mg/kg Nigella Sativa Nanoparticles, A. fat; Abdominal fat, DM; dry matter, CP; crude protein, CF; crude fat.

### Nutrient digestibility

Supplementation with Nano-NS and avilamycin showed effects on nutrient digestibility in broilers under hot climatic conditions (Table 2). Adding Nano-NS and avilamycin significantly increased (*P* < 0.05) dry matter digestibility compared to the control group, while crude fat digestibility was not affected by the experimental additives. However, compared to the other group, significantly increased (*P* < 0.05) crude protein digestibility in broilers fed with a diet including 40 and 50 mg/kg Nano-NS.

### Serum lipid profile and antioxidant status

Increased serum high-density lipoprotein cholesterol (HDL) levels (*P* < 0.05), while lower total cholesterol (TCH) and low-density lipoprotein cholesterol (LDL) concentrations in broilers receiving Nano-NS than those receiving the avilamycin and control diet, as shown in Table 3. Nevertheless, no differences (*P* < 0.05) in serum glucose (GLU), and triglyceride (TRG) levels between experimental groups. Regarding the effect of additives on the oxidation status, increased superoxide dismutase (SOD) levels (*P* < 0.05), while decreasing malondialdehyde (MDA) levels in broilers receiving Nano-NS compared to those receiving the avilamycin and control diet. Additionally, glutathione peroxidase (GPx) levels were not affected (*P* < 0.05) by experimental supplements (Table 4).

**Table 3:** Effect of supplementation of Nano-NS on the immune organs (%), serum immunoglobulins and inflammatory factors (pg/mL) of broilers under high ambient temperature.

Item	Parameter	CON	AVI	Nano-NS_1_	Nano-NS_2_	Nano-NS_3_	SEM	*P*-value
Immune organs	Bursa	2.75^b^	2.68^b^	2.73^b^	2.99^ab^	3.20^a^	0.518	0.020
	Thymus	2.06^c^	2.14^b^	2.01^c^	2.31^a^	2.19^b^	0.166	0.001
	Spleen	1.12	1.05	1.16	1.10	1.18	0.521	0.104
Immunoglobulin	IgM (ng/mL)	12.5	12.9	11.7	12.4	12.8	0.080	0.258
	IgA (ng/mL)	247^b^	252^b^	256^b^	278^a^	281^a^	1.261	0.020
	IgG (µg/mL)	2.35^b^	2.31^b^	2.42^b^	2.67^a^	2.63^a^	0.013	0.011
Inflammatory factors	IL-10	37.9	34.0	35.2	36.5	40.1	0.064	0.055
	IL-6	88.6^a^	75.5^b^	76.2^b^	69.1^c^	62.3^c^	1.825	0.001
	TNF-α	247^a^	240^a^	225^b^	192^c^	188^c^	0.331	< 0.001

^a–c^ Means with different superscripts within the same row differ significantly (*P* < 0.05); SEM standard error of means; CON; basal diet without added, AVI; basal diet with avilamycin, Nano-NS1; basal diet with 30 mg/kg Nigella Sativa Nanoparticles, Nano-NS2; basal diet with 40 mg/kg Nigella Sativa Nanoparticles, Nano-NS3; basal diet with 50 mg/kg Nigella Sativa Nanoparticles, IL-10; interleukin-10, IL-6; interleukin-6, TNF-α; tumor necrosis factor-alpha.

**Table 4:** Effect of supplementation of Nano-NS on the serum lipid profile (mg/dL) and antioxidant status of broilers under high ambient temperature.

Item	parameter	CON	AVI	Nano-NS_1_	Nano-NS_2_	Nano-NS_3_	SEM	*P*-value
Lipid profile	GLU	175.6	178.7	177.9	179.2	170.4	0.518	0.183
	TRG	26.4	25.9	26.6	26.1	25.7	3.132	0.096
	TCH	144^a^	148^a^	139^a^	127^b^	124^b^	2.351	0.094
	LDL	61.2^a^	59.3^a^	60.7^a^	58.4^ab^	56.5^b^	4.115	0.018
	HDL	71.6^c^	72.2^c^	73.1^c^	78.5^b^	82.1^a^	1.055	< 0.001
Antioxidant status	SOD (U.ml^− 1^)	126.5^c^	131.1^b^	129.6^b^	135.7^a^	136.3^a^	0.032	0.010
	MDA(nmol.ml^− 1^)	1.715^a^	1.130^b^	1.242^b^	0.890^c^	0.865^c^	0.951	0.021
	GPx (U.ml^− 1^)	33.5	32.9	33.7	33.4	34.1	1.135	0.102

^a–c^ Means with different superscripts within the same row differ significantly (*P* < 0.05); SEM standard error of means; CON; basal diet without added, AVI; basal diet with avilamycin, Nano-NS1; basal diet with 30 mg/kg Nigella Sativa Nanoparticles, Nano-NS2; basal diet with 40 mg/kg Nigella Sativa Nanoparticles, Nano-NS3; basal diet with 50 mg/kg Nigella Sativa Nanoparticles; GLU; glucose, TRG; triglycerides, TCH; total cholesterol, HDL; high-density lipoprotein, LDL; low-density lipoprotein, MDA; malondialdehyde, SOD; superoxide dismutase, GPx; glutathione peroxidase.

### Immune and inflammatory response

In comparison between the experimental groups, a significant decrease in serum IL-6, and TNF-α was observed in chickens fed on Nano-NS compared to the other groups, in contrast, IL-10 levels were not affected by the experimental additions at 35 d of age (Table 3). Serum IgG and IgA were significantly higher (*P* < 0.05) in chickens receiving Nano-NS than the other groups, as noted in Table 3. However, serum IgM levels were not affected by the experimental treatments. Moreover, dietary Nano-NS supplementation increased (*P* < 0.05) the relative weight of the thymus, and bursa of Fabricius compared with avilamycin and the control groups, while not affecting (*P* < 0.05) the relative weight of the spleen.

### Cecal microflora

Adding dietary Nano-NS or avilamycin had effect different significant (*P* < 0.05) effects on the populations of *Lactobacillus*, Total Coliforms, *C. perfringens*, *Enterobacteriaceae*, and *E. coil* in cecal, as shown in Table 5. Significant increase in the *Lactobacillus* population and a decrease in the *E. coli* population in the chickens (*P* < 0.05) that received the Nano-NS, in addition to slightly reduced *C. perfringens* with an increased level of Nano-NS in the diet. However, reduced *E. coli* and *Lactobacillus* populations, as well as, *C. perfringens* in chickens that received avilamycin. Despite this, supplemented Nano-NS or avilamycin did not affect the cecal microbial populations, such as *Enterobacteriaceae*, and Total Coliforms compared with the control group.

**Table 5:** Effect of supplementation of Nano-NS on microflora in the cecal of broilers under high ambient temperature.

Item	CON	AVI	Nano-NS_1_	Nano-NS_2_	Nano-NS_3_	SEM	*P*-value
Lactobacillus	6.31^b^	6.25^b^	6.83^b^	7.36^a^	7.32^a^	0.208	0.010
Total Coliform	7.05	7.12	7.34	7.15	7.22	0.116	0.337
Enterobacteriaceae	5.83	5.67	5.91	5.85	5.70	0.243	0.510
C. perfringens	2.09^a^	1.45^b^	2.11^a^	1.91^a^	1.51^b^	0.440	0.036
E. coli	4.27^a^	3.16^b^	3.94^a^	3.20^b^	2.84^c^	0.271	0.018

^a–c^ Means with different superscripts within the same row differ significantly (*P* < 0.05); SEM standard error of means; CON; basal diet without added, AVI; basal diet with avilamycin, Nano-NS1; basal diet with 30 mg/kg Nigella Sativa Nanoparticles, Nano-NS2; basal diet with 40 mg/kg Nigella Sativa Nanoparticles, Nano-NS3; basal diet with 50 mg/kg Nigella Sativa Nanoparticles, *E. coli; Escherichia coli*,* C. perfringens; Clostridium perfringens*.

### Ileum gene expression

**Fig. 1:**
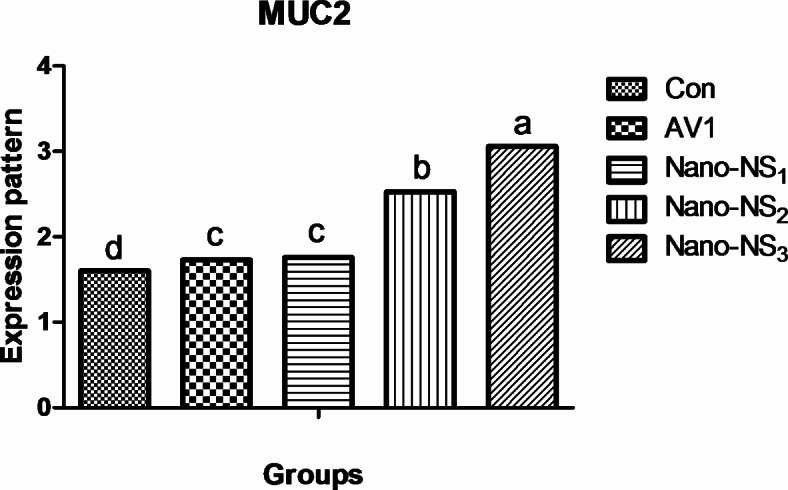
Effects of supplementation of Nano-NS on MUC2 genes expression in the ileum mucosa of broilers. CON; basal diet without added, AVI; basal diet with avilamycin, Nano-NS1; basal diet with 30 mg/kg Nigella Sativa nanoparticles, Nano-NS2; basal diet with 40 mg/kg Nigella Sativa nanoparticles, Nano-NS3; basal diet with 50 mg/kg Nigella Sativa nanoparticles. ^a–c^ Mean value above each bar with no common superscript differs significantly (*p* < 0.05). Error bars represent SEM.

The effect of adding Nano-NS on gene expression in the ileum is shown in Fig. 1. Relative expression of the MUC2 gene was upregulated in the broilers fed 50 mg/kg Nano-NS (*P* < 0.05) compared with other and control groups at 35 d.

## Discussion

Previous studies have confirmed that using feed additives is necessary to mitigate the negative effects of HS on broilers raised in hot climates^10,24^. Therefore, our study investigated the potential positive role of Nano-NS supplementation in mitigating the effects of heat stress by enhancing the growth performance and health of broiler chickens.

Growth performance is the key indicator for evaluating the impact of feed additives on economic benefits and production performance. As we expected, the addition of Nano-NS in this study resulted in a significant improvement in BWG and FCR compared to that of the avilamycin and control groups. Our results were supported by many reports that found an improvement in growth performance in the chicken fed a diet of nigella seed oil^25,26^. Moreover, supplementing with crushed nigella sativa seeds significantly increased body weight and decreased the FCR compared with the control group^27^. Furthermore, the BWG of the broiler increased significantly in groups supplemented with a nigella seed oil diet compared with the control group^28^. On the other hand, Denli et al.^26^ reported that dietary black cumin seed extract supplementation did not affect feed intake in quail. As well, Mohammed and Al-Suwaiegh^29^ reported that adding NS to the diet stimulated thyroid hormone secretion via the pituitary gland, which enhanced the metabolic rate and better amino acid use. Furthermore, in the current study, chickens receiving the Nano-NS supplementation showed a significant increase in EPEF value (based on FCR, LBW, and mortality (%)), which indicates improved growth performance. Subsequently, the improvement in growth performance (BWG and FCR) of birds receiving Nano-NS, although the FI was not affected, can be explained by increased nutrient digestibility and increased digestive enzyme activity^8,25^ thus improving feed utilization efficiency. Additionally, it has antimicrobial and antioxidant properties^25,30^. Therefore, the addition of Nano-NS has a positive role in enhancing growth performance while the broiler is exposed to HS.

Our results showed a significant increase in the dressing percentage in birds fed Nano-NS compared with the other groups, while the relative weight of the thigh, breast, liver, and abdominal fat were not affected in this study. Our results were similar to those of Toghyani et al.^31^, who found an increased carcass yield in broilers fed a diet containing black seed. Likewise, many studies confirmed a significant improvement in the dressing percentage and relative weight of the liver (68.92–72.78%) in chickens fed NS^32^. The increase in the dressing percentage can be attributed to the noticeable improvement in growth performance (BWG and FCR) through the antimicrobial role (inhibit pathogens in the digestive system) in enhancing nutrient utilization and gut health^33^. In addition, our results are similar to Hermes et al.^32^ who found no significant effect on abdominal fat and giblet percentage by feeding different levels of *N. Sativa* seed in broilers. From this, the beneficial role of Nano-NS-supplements in enhancing carcass characteristics can be clarified.

In this study, broiler diets supplemented with Nano-NS resulted in an increase in the digestibility of crude protein and dry matter. Similarly, adding essential oils to the broiler feed improved the digestion of crude protein, fat, and cellulose^8,34^. Herbs and their products (such as essential oils) are incorporated into the chicken diet to stimulate more effective use of feed nutrients by increasing enzyme activity, which leads to improving nutrient digestibility, which is reflected in the enhanced performance in this study^25,35,36^. Furthermore, many studies have shown the beneficial impact of dietary inclusion of essential oil on growth performance via increasing trypsin and pancreatic amylase activity^37,38^. This effect could be related to the ability of Nano-NS to enhance enzyme activity in the digestive system^8,12^ and modify the microbial content of the gut^13^, which boosts nutrient metabolism.

Consumers recently have been interested in knowing foods that are low in cholesterol because they have a beneficial effect on their health, which reduces the risk of atherosclerosis and coronary artery diseases. Poultry meat is characterized by its low cholesterol when compared to other meat sources. For this purpose, the effect of Nano-NS on the lipid profile was investigated in this study. The findings in the current study of the broiler serum lipid profile were consistent with prior studies of the effects of essential oils, where there was a significant decrease in the level of cholesterol and LDL, while the level of HDL increased in broilers fed supplement Nano-NS. Similar results were reported by AL-Beitawi et al.^39^ who noted that black cumin addition reduced cholesterol concentration and increased HDL concentrations in broilers plasma. Similarly, EL-Bagir et al.^40^ found decreased serum phospholipid and cholesterol levels in laying hens fed a diet containing 1.5% black cumin. Furthermore, Akhtar et al.^41^ reported that the inclusion of 1.5% black cumin markedly decreased cholesterol in the yolk (227 to 199 mg/egg). The significant changes in blood lipid levels in the current study are due to the active biological ingredients (thymoquinone) and compounds like monounsaturated fatty acids that decrease the fractional absorption of cholesterol from the small intestine and lower the cholesterol synthesis by hepatocytes^13^. In addition, the current study revealed that supplementing with Nigella sativa nanoparticles in broiler diets resulted in a higher concentration of HDL. It is known that the high level of HDL in the blood has a useful effect on the bird’s body and conditioning the transport of cholesterol from the peripheral tissue to the liver^24^. The current study demonstrates the effective role of Nano-nigella sativa supplements in enhancing the lipid profile in the blood of broilers exposed to HS, which enhances the health of the bird and the consumer.

Exposure of chickens to HS leads to an imbalance between the rate of free radical production and the body’s biological oxidation system, which is known as oxidative stress^42^. Using natural antioxidants in poultry feed is an important addition to control fat oxidation, thus preventing some diseases, which enhances the bird’s health^21^. This attracted the attention of nutritionists to add some herbs or their products to poultry feed because they contain some biologically active compounds that have an affected role as a natural antioxidant^9,43^. It is important to measure the levels of oxidative enzymes during HS, especially SOD because of their importance in converting superoxide free radicals into molecular oxygen and hydrogen peroxide to mitigate the effects of HS on birds^44^. In our study, it was observed that dietary supplementation of Nano-NS in the broilers’ diet led to a reduction in the MDA level and an increase in the SOD level when compared to avilamycin and the control groups. This conforms with the results of Ahmad et al.^45^, who found an increase in antioxidative enzyme activity in broilers feeding on nigella seed oil. This conclusion is consistent with the data reported by Tuluce et al.^46^ adding black cumin to the diet of broilers led to MDA levels significantly decreasing. Similar to our study, Guler et al.^47^ reported that MDA levels in all tissues were considerably lower in all the black cumin seed-treated groups than in the control group. The study carried out on broilers showed that black cumin increased the activities of several enzymes such as glutathione-S-transferase, catalase, and adenosine deaminase, which resulted in reducing oxidative stress in the liver^48^. From our results, it can be suggested to add Nano-NS as a cytoprotective agent against tissue damage and as a natural potential antioxidant promoter for chickens exposed to HS.

Immune function is affected by many factors including diet composition, environment, stress, etc. This responsiveness to external influences has led to many efforts to enhance immune function through manipulating nutrition (nutritional immunomodulation) to reduce or eliminate specific pathogens. Immune organs (Spleen, thymus, and bursa of Fabricius) for poultry are closely associated with immune functions and greater weights of lymphoid organs usually represent stronger immune functions to some extent^49^. Furthermore, lymphoid tissue plays a role in generating antibodies that stimulate immunological responses^50^. In the present study, the dietary supplementing of Nano-NS led to elevating weights of the thymus, and bursa of Fabricius, which is in agreement with the report of Bayati et al.^51^, who found that the broiler’s diet supplemented with salvia essential oil led to increased weights of the bursa of Fabricius. The increase in the relative weight of the thymus and bursa of Fabricius might be due to enhancing proliferation of immune cells in primary lymphoid organs, which represents better immunity, as a result of the beneficial role of the bioactive compounds in Nano-NS and the effect on the functional activities of the immune system which led to an enhancement in the immune responses of the birds^52^. Therefore, Nano-NS supplements can be used as an immune stimulant while the bird is exposed to stress.

The antioxidant and anti-inflammatory characteristics of essential oil and other plant extracts support their use as nutritional supplements in broiler feed to directly or indirectly affect the immune response. Many studies have confirmed the importance of maintaining intestinal integrity due to its main role in supporting the bird’s health, which is represented in immune defense and antigen resistance, which is detected through some procedures such as evaluating the concentrations of cytokines and immunoglobulin 53. The main molecule that affects the intestinal immune response system is IgA which can prevent viruses, bacteria, or some harmful antigens from adhering to the intestinal epithelial, thereby promoting cellular and intestinal immunity^54^. Moreover, IgG is the major immunoglobulin subclass and plays a vital role in inactivating multiple immune effector systems, it is secreted by B cells^53,54^. The current study results showed a decrease in levels of IgA and IgG in chickens fed diets without feed additives under environmental heat stress. Several studies have shown a significant decrease in immunoglobulin levels in chickens exposed to HS^55^. Furthermore, in this study, IgA and IgG levels increased in the serum of broilers received the Nano-NS implying that there was inflammation due to pathogen invasion, as a result of heat stress. A previous report has shown that the change in gut permeability after HS is amplified by inflammation because the loss of gut permeability after stress allows the entry of pathogenic bacteria to the lamina propria, causing local inflammation^56^. The active compounds in essential oils regulate the gut microbiota, which closely interacts with the host’s immune system^8,57^. Similar to our results, Liu et al.^58^ presented that supplementing with essential oils increased SIgA gene expression in the intestine, which maintains intestinal integrity in broilers. Like this, essential oil supplementation in the diet increased the blood levels of IgA, IgM, and IgG in broilers^59^. This shows that supplementation of Nano-NS incited the production of immune responses, resulting in an increase in the IgA secretions. Nano-NS may induce the morphological and functional activation of mononuclear macrophages, leading to enhancing the immune level of birds. From the above, the immunological role of essential oil additives (such as Nano-NS) is clear by increasing immunoglobulin levels in broiler chickens under HS.

The inflammatory cytokines, such as TNF-α, IL-6, and IL-10, are mainly implicated in the inflammatory response^58^. TNF-α and IL-6 are the most important inflammatory mediators that appear in the process of the inflammatory response (produce this pro-inflammatory cytokine). The balance between proinflammatory cytokines and anti-inflammatory is an essential factor in immune responses. Surprisingly, in the current study, broilers that were exposed to hot climatic conditions and received the Nano-NS showed reduced IL-6, and TNF-α concentrations in the serum of broilers compared to other groups, which indicates that it enhanced immune function and reduced the inflammatory response in broilers. In agreement with the present study, increased IL-4 and IL-10 levels and decreased TNF-α and IL-1β levels in the serum of broilers fed a diet containing essential oil^59^. This conforms with the results of Yadav and Chandra^60^, who found decreased levels of TNF-α and IL-1β in broilers feeding on the essential oil. The results of the present study indicate that Nano-NS supplementation has anti-inflammatory roles in heat-stressed broiler chickens.

Several studies have confirmed that there is an association between metabolic disorders, structures of the intestinal microflora, and broiler health under HS^2,61^. The present study found that adding Nano-NS during HS enhances the colony composition in cecal contents. The results of the current study showed a decrease in the *E. coli* and *C. perfringens* count and an increase in the *Lactobacillus* count. The results of the present study correspond with those of studies reporting the addition of essential oils that had decreased *E. coli* populations^62^. Consistent with the results of this report, Pham et al.^63^ and Yilmaz and Gul^64^ have recently confirmed essential oils’ and aromatic herbs potential to modulate the gut bacterial community structure, which improves gut health. It is believed that the effect of Nano-NS as an antimicrobial on the intestinal microflora is due to the effect of biologically active compounds in stimulating the production of some short-chain fatty acids (SCFA) that have an important role in gut bacteria structures. The essential oils showed enhanced ceca acetic, butyric, propionic, and lactic acids and total SCFA concentration, which serves as an important energy substrate for the maintenance and proliferation of gut cells and structures in broiler chickens^65,66^. Regarding the results of the present study, it seems that in hot climatic conditions, Nano-NS significantly affected the intestine’s microbial population, improving gut and bird health. In summary, the results of the present study suggest that supplementing with Nano-NS improved gut health by boosting anti-inflammatory cytokines and antimicrobial properties, thus improving broiler growth performance under hot climatic conditions.

To evaluate the HS impact, many parameters have been used; however, expression profiling of genes may play pivotal roles during exposure to HS. Different defensive activities are stimulated to protect the cells of tissues during stress, including the expression of stress response gene coding, like the mucin 2 gene (MUC2). HS in broilers leads to suppressing the immune system by regulating the expression of genes such as cytokines regulation or ileum mucin, which are important markers of immune and nutrient-absorbed regulation^67^. Thus, the regulation of related genes under HS can act as a marker to determine the extent to which the bird is exposed to stress. In the current study, HS led to decreasing ileum MUC2 gene expression, thus a negative impact on the nutrient-absorbed system. However, in the present study, adding Nano-NS led to increasing regulation of MUC2 gene expression. MUC2 is the main mucin produced by cuprocytes and is a significant component of the mucus layer covering the intestinal epithelium. Additionally, mucin is the major constituent of the mucus layer and serves a definitive role in protecting the intestinal from digestive enzymes, acidic chyme, and pathogens^68^. Similar results were obtained, an increase in jejunum mucin 2 gene expression in the jejunum in broilers fed a diet that includes essential oil^69,70^. Bioactive compounds regulate mucin 2 gene expression by altering the activity of transcription factors such as Fox1 and GATA4 which play an important role in regulating some gene expression^71^. In addition, the antimicrobial properties of essential oil could help catalyze the growth of small intestinal mucosal absorptive cells. The difference in their expression of the barrier gene may be related to the ileum bacterial species, in which it has been shown that supplements can modify the microbial content in the intestine, thus increasing the expression of the MUC2 gene in broiler chickens^72^. It has also been found that increased *Lactobacillus* count in jejunum significantly increased the expression of the MUC2 gene in broilers^73^. Certain *Lactobacilli* attenuate barrier disruption by up-regulating some genes. Moreover, *Lactobacillus* increases the expression of closure proteins^74^ and improves the integrity of the intestinal barrier^75^. Our results show a positive impact on gut health through modulating microbial content and regulating gene expression, thus enhancing digestion and absorption of nutrition and growth performance in broilers under high ambient temperatures.

## Conclusions

Dietary Nano-NS supplementation improves productive performance by enhancing the growth, carcass characteristics, and nutrient digestibility of broilers under high ambient temperatures. In addition, Nano-NS supplementation showed an effective effect in enhancing the immune response, antioxidant status, and gut health by modulating microbial content and regulating the gene expression of MUC2 of broiler chickens under environmental heat stress. Therefore, Nano-NS supplementation had effective impacts in promoting the health of heat-stressed broiler chickens and may serve as a useful nutritional strategy for anti-heat stress.

## Materials and methods

### Experimental design and birds management

**Fig. 2:**
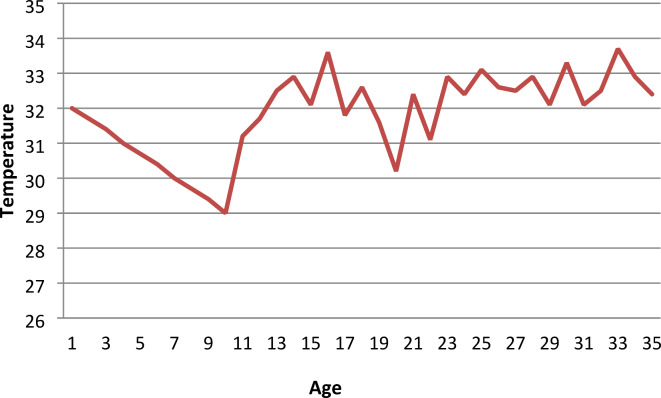
Temperatures during the trial period.

The trial was conducted on three hundred and seventy-five male broiler chicks (1-day-old Ross-308) with a similar body weight (41.6 ± 0.3 g) obtained from a commercial hatchery. Chicks were randomly allocated to five experimental groups with 5 replicates (15 chicks for each replicate). The experimental groups were as follows: the first group was fed a basal diet with no additives (control group, CON), and the second group was fed the basal diet supplemented with avilamycin at a level of 50 mg/kg (AVI), while the third, fourth, and fifth groups were fed the basal diet supplemented with 30, 40, and 50 mg/kg Nano-NS, respectively (Nano-NS1, Nano-NS2, and Nano-NS3). Chicks were fed two based basal diets (corn-soybean) for 35 d divided into two stages: the first stage (starter, 1 to 21d), the second stage (grower, 22 to 35d), as presented in Table 6. Diets were formulated to satisfy the nutritional requirements according to the National Research Council (NRC^76^). All broiler chicks were grown in metal cages with food and fresh water provided ad libitum. The temperature was set at 32 °C for the first two days, afterward, the temperature was gradually reduced to 29 °C until the tenth day of the experiment, then the birds were raised at ambient temperature during the summer from 11 days of age until the end of the experiment. Temperature and humidity were recorded twice a day at 1 pm and 1 am until the end of the experiment. The relative temperature ranged from 30.2 °C to 33.7 °C, (Fig. 2) and average relative humidity 56% throughout the experimental period. During the first five days, the chicks were exposed to 24 h of light per day, then reduced to 22 h from 6 to 10 days of age, and eventually reduced to 20 h per day until the end of the experiment. Experimental broilers among all groups were in good health throughout the experimental period of 35 days. Cold-pressed Nigella sativa oil was analyzed by using gas chromatography-mass spectrometry (GC–MS), shown in Table 7. Nigella sativa nanoparticles were obtained from the Nanotechnology Laboratory at the National Research Center in Egypt.

**Table 6:** Composition of experimental diets.

Ingredient (%)	Starter (0-21d)	Grower (22-35d)
Yellow corn	55.40	59.20
Soybean meal	38.06	33.10
Corn Oil	2.380	4.050
Di-Calcium Phosphate	2.040	1.820
Calcium carbonate	1.270	1.060
Premix*	0.300	0.300
Salt	0.250	0.250
DL-Methionine	0.160	0.120
Hcl-Lysine	0.040	-
Sodium bicarbonate	0.100	0.100
Total	100	100
Chemical composition		
Crude protein (%)	23	21
Metabolizable energy(kcal/kg)	3000	3200
Calcium (%)	1.045	0.941
Available Phosphorus (%)	0.497	0.451

*Premix: (1%) provided the following (per Kilogram of complete diets). 1400 IU Vitamin A, 3000 IU Vitamin D3, 50 mg Vitamin E, 4 mg Vitamin K, 3 mg Vitamin B6, 6 mg Vitamin B12, 60 mg Niacin, 20 mg Pantothenic acid, 0.20 mg folic acid, 150 mg Choline, 48 mg Ca, 3.18 mg P, 100 mg Mn, 50 mg Fe, 80 mg Zn, 10 mg Cu, 0.25 mg Co, 1.5 mg Iodine.

**Table 7:** Chemical composition for cold-pressed Nigella sativa oil.

Compound	% of total
Caryophyllene	19.47
Thymoquinone	16.80
1,4-Cyclohexadiene	8.03
p-cymene	6.27
Longifolene	4.5
Carvacrol	2.16

### *Nigella sativa* nanoparticles (Nano-NS) preparation

Nigella sativa oil (cold-pressed black cumin oil) was purchased from Taiba Aromatic and Medicinal Products Company to prepare the *Nigella sativa* nanoemulsion in the Nanotechnology Laboratory at the National Research Center, Egypt. The ionic gelation method was used as described by Koukaras et al.^77^. The shape and size of Nano-NS were measured by transmission electron microscopy to estimate the distribution of different-sized particles dispersed in Nano-NS solution and its stability according to Abdelhakim et al.^78^. After obtaining the Nano, it was stored at 4 °C until added to the broiler experimental diet.

### Growth performance and carcass characteristics

Growth performance of the birds including the live body weight (LBW), body weight gain (BWG), feed intake (FI), and feed conversion ratio (FCR = FI (g)⁄ BWG (g)) was measured on week 3 and week 5. In addition, the Mortality rate and the European Production Efficiency Factor (EPEF = (Livability (%)× LBW (g))/(age (days)× FCR)× 100) were calculated. On day 35, six chicks/groups were randomly selected to slaughter (euthanasia) for evaluating carcass characteristics. Dressing (%), and relative weight of thigh, breast, liver, abdominal fat, and immune organs (bursa of Fabricius, thymus, and spleen as immune indices) were calculated.

### Digestibility trial

Five broilers from each group were separated and placed individually in digestion cages at 35 days of age. The digestion experiment lasted for 4 days. Fresh excreta samples were collected from beneath each bird every 8 h daily during the 4-day digestion experiment and then dried. Additionally, the amount of feed intake during the digestion period was recorded to measure nutrient digestibility coefficients. The feed and excreta samples collected were analyzed at the Desert Research Center Laboratory in Egypt for dry matter (DM), crude protein (CP), and crude fat (CF) using the methods of AOAC^79^.

### Serum chemistry

At the end of the experimental period, 5 chicks from each group to blood samples, from the wing vein, which were gathered in non-heparinized tubes to get the serum. Serum was obtained by centrifugation at 4,000×g for 15 min at 4 °C and the serum was harvested and stored at -20 °C until analyses. Serum concentrations of glucose (GLU), triglycerides (TRG), total cholesterol (TCH), high-density lipoprotein (HDL), and low-density lipoprotein (LDL) were determined using commercial kits spectrophotometrically (Spectronic 1,201, Milton Roy, Ivyland, PA, USA). Additionally, assays of malondialdehyde (MDA), superoxide dismutase (SOD), and glutathione peroxidase (GPx) were performed using commercial kits (BioAssay Systems, USA and Cayman Chemical Company, USA). Levels of immunoglobulin G (IgG), Immunoglobulin A (IgA), and Immunoglobulin M (IgM) in serum were estimated using chicken-specific immunoglobulin ELISA quantitation kits (Bethyl Laboratories Inc., Montgomery, TX, USA). Blood samples were used for detecting concentrations of cytokines of interleukin-6 (IL-6), interleukin-10 (IL-10), and tumor necrosis factor-a (TNF-a) using commercial ELISA kits (MyBioSource, San Diego, CA). According to the manufacturer’s instructions, all screening procedures were performed.

### Cecal microflora

During the slaughter at 35 d, the contents of the cecal were collected. One gram of the sample was taken into sterile glass containers, then diluted 1:10 in 9 ml Ringer’s diluent (pH 6.8∼7.2) and homogenized. Then, 1 mL of dilutions was spread on appropriate selective agar media for enumeration for each microbe under study. Bacterial colonies were counted by the pour plate method. Each microbe was grown under special conditions of temperature and an appropriate environment. *Lactobacillus* (MRS agar, Merck, Darmstadt, Germany), Total Coliform and *Enterobacteriaceae* (VRBD agar, Merck, Darmstadt, Germany), *Clostridium perfringens* (SIA agar, Merck, Darmstadt, Germany), and *Escherichia coli* (deMan agar, Merck, Darmstadt, Germany) were estimated. Using the traditional method of microbial enumeration of cecal contents (diffusion plate method), microbial enumeration was performed as described by Abdel Moneim et al.^80^.

### Gene expression

According to the reported methods by Yang et al.^62^, RNA extraction from the ileum mucosa using RNAiso Plus reagent (Takara, China) and the procession of reverse transcription and real-time PCR were performed. cDNA was synthesized using A Superscript™ II Reverse Transcriptase kit (Invitrogen, Carlsbad, USA), and then cDNA was diluted to 10 ng/uL for qRT-PCR analysis. Using the 2– ^ΔΔCt^ method, the relative expression level of the mucin 2 (MUC2) gene was calculated by gene expression normalized to b-actin. The forward and reverse primers for mucin 2 were AACTCCTCCTTTGTATGCG and ATTCAACCTTCTGCCCTAA; for βeta-actin: GAGAAATTGTGCGTGACATCA and CCTGAACCTCTCATTGCCA.

### Statistical analysis

All data were analyzed by one-way ANOVA using the Statistical Analysis System (SAS Institute^81^) followed by Duncan’s multiple range test. Statistical differences among group means were considered significant at *p* < 0.05.

## Data Availability

The datasets generated during and/or analyzed during the current study are available from the corresponding author on request.

## Abbreviations

BWG: Body weight gain
*C. perfringens*: *Clostridium perfringens*
CF: Crude fat
CP: Crude protein
DM: Dry matter
*E. coli*: *Escherichia coli*
FCR: Feed conversion ratio
FI: Feed intake
GLU: Glucose
GPx: Glutathione peroxidase
HDL: High-density lipoprotein
HS: Heat stress
IL-10: Interleukin-10
IL-6: Interleukin-6
LBW: Live body weight
LDL: Low-density lipoprotein
MDA: Malondialdehyde
MUC2: Mucin 2
Nano-NS: *Nigella sativa* nanoparticles
SAS: Statistical analysis system
SOD: Superoxide dismutase
TCH: Total cholesterol
TNF-α: Tumor necrosis factor-alpha
TRG: Triglycerides
